# A Landscape Epidemiological Approach for Predicting Chronic Wasting Disease: A Case Study in Virginia, US

**DOI:** 10.3389/fvets.2021.698767

**Published:** 2021-08-24

**Authors:** Steven N. Winter, Megan S. Kirchgessner, Emmanuel A. Frimpong, Luis E. Escobar

**Affiliations:** ^1^Department of Fish and Wildlife Conservation, Virginia Tech, Blacksburg, VA, United States; ^2^Virginia Department of Wildlife Resources, Blacksburg, VA, United States; ^3^Global Change Center, Virginia Tech, Blacksburg, VA, United States; ^4^Center for Emerging Zoonotic and Arthropod-borne Pathogens, Virginia Tech, Blacksburg, VA, United States

**Keywords:** chronic wasting disease, CWD, wildlife disease, landscape epidemiology, prion, hypervolume

## Abstract

Many infectious diseases in wildlife occur under quantifiable landscape ecological patterns useful in facilitating epidemiological surveillance and management, though little is known about prion diseases. Chronic wasting disease (CWD), a fatal prion disease of the deer family Cervidae, currently affects white-tailed deer (*Odocoileus virginianus*) populations in the Mid-Atlantic United States (US) and challenges wildlife veterinarians and disease ecologists from its unclear mechanisms and associations within landscapes, particularly in early phases of an outbreak when CWD detections are sparse. We aimed to provide guidance for wildlife disease management by identifying the extent to which CWD-positive cases can be reliably predicted from landscape conditions. Using the CWD outbreak in Virginia, US from 2009 to early 2020 as a case study system, we used diverse algorithms (e.g., principal components analysis, support vector machines, kernel density estimation) and data partitioning methods to quantify remotely sensed landscape conditions associated with CWD cases. We used various model evaluation tools (e.g., AUC ratios, cumulative binomial testing, Jaccard similarity) to assess predictions of disease transmission risk using independent CWD data. We further examined model variation in the context of uncertainty. We provided significant support that vegetation phenology data representing landscape conditions can predict and map CWD transmission risk. Model predictions improved when incorporating inferred home ranges instead of raw hunter-reported coordinates. Different data availability scenarios identified variation among models. By showing that CWD could be predicted and mapped, our project adds to the available tools for understanding the landscape ecology of CWD transmission risk in free-ranging populations and natural conditions. Our modeling framework and use of widely available landscape data foster replicability for other wildlife diseases and study areas.

## Introduction

Effective wildlife disease management and control depends upon epidemiological surveillance, though identifying geographic locations where surveillance should be deployed can be challenging and require extensive sampling regimes (1). More recent advances in disease ecology and biogeography have identified likely areas for pathogen presence from associations between disease occurrence and landscape characteristics using correlative methods (2, 3). Comprehensive protocols and conceptual bases in landscape epidemiology have been well-developed for diseases affecting humans and domestic animals (4) but are still in development for wildlife (5). For example, before the early 2010s, landscape epidemiology approaches remained generally limited for prions (6). Prions are a group of infectious pathogens that cause neurodegenerative diseases in humans and animals (7). This perceived lag may be the result, at least in part, to unclear origins of prion biology and the atypical biological properties of prions with respect to other pathogens (8). Moreover, because of their inextricable connection with hosts and the unclear role of other animals in their propagation, prion diseases remain a unique challenge in wildlife epidemiology (9).

Chronic wasting disease (CWD) is a prion disease of wildlife (10). Identified in wild cervid populations in the western United States (US) since the 1980s (11), CWD was not detected in eastern portions of the US until the early 2000s (12). High CWD prevalences have been shown to diminish wild cervid population viability (13, 14); therefore, monitoring and surveilling for the highly contagious and invariably fatal disease is crucial for wildlife management. Direct contact between susceptible and infected cervids can transmit prions causing CWD (15, 16). Also, prion contamination of the landscape through infected hosts' bodily fluids and tissues (9, 17) can indirectly transmit the pathogen and complicate CWD control.

Most modeling efforts to reconstruct and predict CWD risk factors related to CWD transmission have prioritized detailed population-level assessments that have generated useful findings for management (6, 18), which have led to unified, formalized guidelines in the US (19). Importantly, recent work with agent-based models have supported the use of management strategies tailored to the phase of the CWD outbreak (20). Still, with respect to population modeling, formal protocols for, and the role of, the landscape has seen less attention in CWD epidemiology. This disparity is probably because of high data demands for many modeling designs (6) and potential for regional or temporal nuance (i.e., stage of the outbreak) to influence conclusions on landscape risk factors and distributional predictions. For example, recent research focused on the CWD cluster in the Mid-Atlantic US (i.e., Maryland, Pennsylvania, Virginia, West Virginia) identified forested landscapes to be negatively related to CWD occurrence (12, 21); this finding contradicted patterns in disease distributions found in the Midwestern US where CWD was found to be positively related to forested landscapes (22, 23). Evans et al. (21) postulated that reduced CWD occurrence in forested landscapes could be a CWD-landscape relationship unique to the Mid-Atlantic or indicative of the early phase of the outbreak, which may suggest that tailoring landscape epidemiological modeling approaches to the phase of the outbreak is warranted. Indeed, most CWD-landscape models rely on contrasting environmental conditions from harvest locations of both CWD-infected and CWD-not detected individuals (6), which could make them sensitive to both diagnostic imperfections and the stage of the outbreak (i.e., zero inflation). Such sensitivities could potentially obscure our understanding of CWD-landscape relationships and predicted distributions.

A black-box modeling framework in landscape epidemiology, as discussed in Johnson et al. (24), is an analysis that aims to detect major patterns or trends in disease phenomena even if the mechanisms causing those patterns cannot be identified. The framework uses locations of known disease cases as occurrence data for model calibration and predicting disease distributions when specific transmission mechanisms are not entirely known and/or uncertainty exists in negative disease results (24, 25), as is the case for CWD whereby positive detections have better reliability (high specificity) than negative results (26, 27). Often, data collected from remote sensing technologies facilitate black-box landscape epidemiology by acquiring environmental variables (e.g., vegetation phenology data) (28) at local scales that may otherwise be unattainable *in situ* or were not collected at the time of disease emergence (29–31). There are three general components of black-box frameworks: the epidemiological data (i.e., occurrence data), landscape data, and the algorithm used to predict distributions (32), which then require model evaluation of predictive performance. A black-box framework is dependent on an algorithm being selected, but the kind of algorithm selected should meet the needs of the disease, data, and researchers' questions. Black-box frameworks rely on the assumption that recorded locations of disease presence possess conditions associated with higher risk for disease transmission, and thus can be used to predict disease distributions. Nevertheless, black-box frameworks are not immune to influence from sampling bias, similar to other landscape models (33–36).

**Figure 1:**
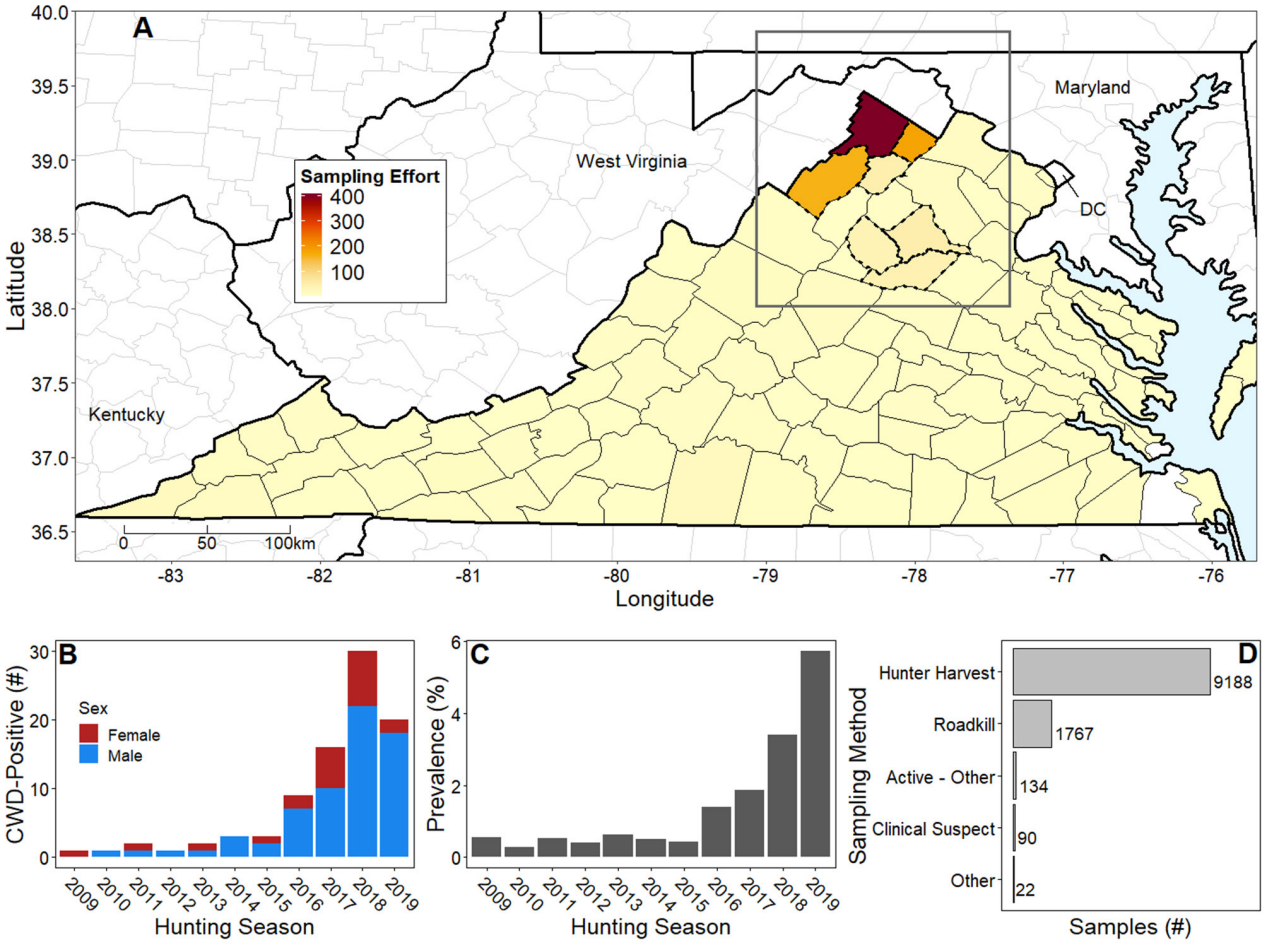
Natural history of chronic wasting disease in Virginia, US from 2007 to early 2020. **(A)** Virginia county colors represent average annual number of deer samples ranging from no samples (white) to highest sampling intensity in Frederick County (dark red; ~400 deer/year); mean cumulative number of samples is 143 white-tailed deer per county. In response to CWD detections, DWR increased sampling intensity and delineated disease management areas (dotted county lines). Our case study area (dark gray rectangle) focused on the northern tip of Virginia and portions of Maryland, Pennsylvania, and West Virginia. **(B)** Stacked bar chart shows sex ratios of CWD-positive cases from 2009 to 2019 hunting seasons. The apparent drop in the number of cases in 2019 is attributed to reallocation of DWR resources to prioritize sampling in non-CWD endemic counties. **(C)** Bar chart shows prevalence in CWD endemic Frederick County from DMA1 increasing over time. Details of higher prevalence values in some regions are lost due to administrative boundaries. **(D)** Horizontal bar chart shows hunter harvest as the predominant sampling method, followed by testing roadkill and clinical suspect cases. Please note that hunting seasons begin in late calendar months (typically November) and extend into early months of the following year.

Presently, CWD is actively spreading in the Mid-Atlantic US and refined guidance on CWD surveillance is critically needed. For example, sustained active and passive surveillance and monitoring effort throughout the state of Virginia has identified increasing CWD burden in recent years (Figure 1). We hypothesized that a black-box landscape epidemiology framework can significantly predict areas suitable for CWD transmission to guide prioritization of surveillance. In this study, we test our hypothesis by utilizing an epidemiological dataset from the state of Virginia and remote sensing vegetation phenology data to identify the extent at which CWD in Virginia can be reliably predicted. In light of sensitivity of landscape models to sampling biases, we examined predictions and model uncertainty under two assumptions from which landscape conditions were determined: (i) observing vegetation phenology variation at precisely reported harvest or sampling locations, and (ii) evaluating phenology variation over a generalized home range scale. With this analysis, we aim to facilitate management decisions (e.g., disease management area delineation), guide CWD surveillance, and assess the utility of a widely accessible remote sensing variable using landscape epidemiology methods to predict a wildlife disease in the early stages of a local outbreak.

## Materials and Methods

### CWD in Virginia

Virginia Department of Wildlife Resources (DWR) began testing free-ranging deer for CWD in 2002; however, active surveillance was not formally initiated until 2005, after a white-tailed deer (*Odocoileus virginianus*) tested positive for CWD in neighboring West Virginia (37). Active, systematic epidemiological monitoring has largely occurred in Virginia's disease management areas, which have been created in response to CWD confirmations (Figure 1A). On the West Virginia border, DWR detected the first CWD-positive deer in Virginia in 2009, necessitating the creation of Disease Management Area 1 (DMA1). Presently, DMA1 located in northwestern Virginia, is delineated by the political boundaries of Frederick, Shenandoah, Clarke, and Warren counties (Figure 1A) (38). Subsequently, DWR has identified increasing CWD incidence over time within DMA1 (Figure 1B) with incidence being defined as the number of new CWD-positive deer. More importantly, however, DWR has documented increasing annual ratios of CWD-positive deer to total deer tested, or prevalence, in localized, CWD-endemic areas (i.e., locations over time within DMAs where DWR determines CWD is established following review of cumulative prevalence and distribution) (37) (Figure 1C), consistent with the early phase of the outbreak (20). Sampling to date has been achieved through diverse sampling methods including active surveillance *via* hunter harvest and roadkill sampling and passive surveillance *via* the testing of clinically ill deer (Figure 1D). In 2019, a second Disease Management Area (DMA2) was developed in Culpeper, Madison, and Orange counties in response to a CWD confirmation in Culpeper County.

### Descriptions of Epidemiological and Landscape Data

By March 2020, DWR reported 88 confirmed cases of CWD in Virginia *via* postmortem extraction of medial retropharyngeal lymph nodes (37) (Figure 1B). These data largely originated from DMA1 hunting grids at 2.59 km^2^ spatial resolution. DWR also confirmed the exact locations for each CWD-positive deer with hunters and other reporting parties to reduce spatial uncertainty. The majority of confirmed cases were collected from hunter harvest; however, other sampling methods (e.g., clinical sign euthanasia, roadkill, etc.; Figure 1D) identified a small number of opportunistic confirmations. Preliminary CWD testing was accomplished *via* using either enzyme-linked immunosorbent assay (ELISA) or immunohistochemistry (IHC) for all samples collected in Virginia. All confirmatory testing was accomplished *via* IHC at the National Veterinary Services Laboratory, Ames, Iowa (37, 39).

We used vegetation indices as surrogates of landscape characteristics across space and time. Vegetation indices are versatile remotely sensed metrics of photosynthetically active radiation and vegetative evapotranspiration that consistently identify and correlate with landscape patterns (40). More specifically, we used the enhanced vegetation index (EVI) due to its strong relationship with vegetative productivity, elevation, temperature, precipitation, and soil characteristics (41, 42), which have associations with CWD distributions. Enhanced vegetation index also corrects for soil and atmospheric interferences, while remaining sensitive to canopy-structured evapotranspiration found in forested land cover types (30, 40, 43). We collected EVI-gridded raster data at 250-m spatial resolution and 16-day temporal resolution from the MODIS sensor in the Terra satellite of NASA using the MODIStsp R package (44, 45). We collected EVI data from 2005 to early 2020, assuming CWD circulation at least 4 years before the first detected case in Virginia (i.e., 2009), which corresponds to the maximum incubation period in white-tailed deer (46).

### Landscape Data Preparation

**Figure 2:**
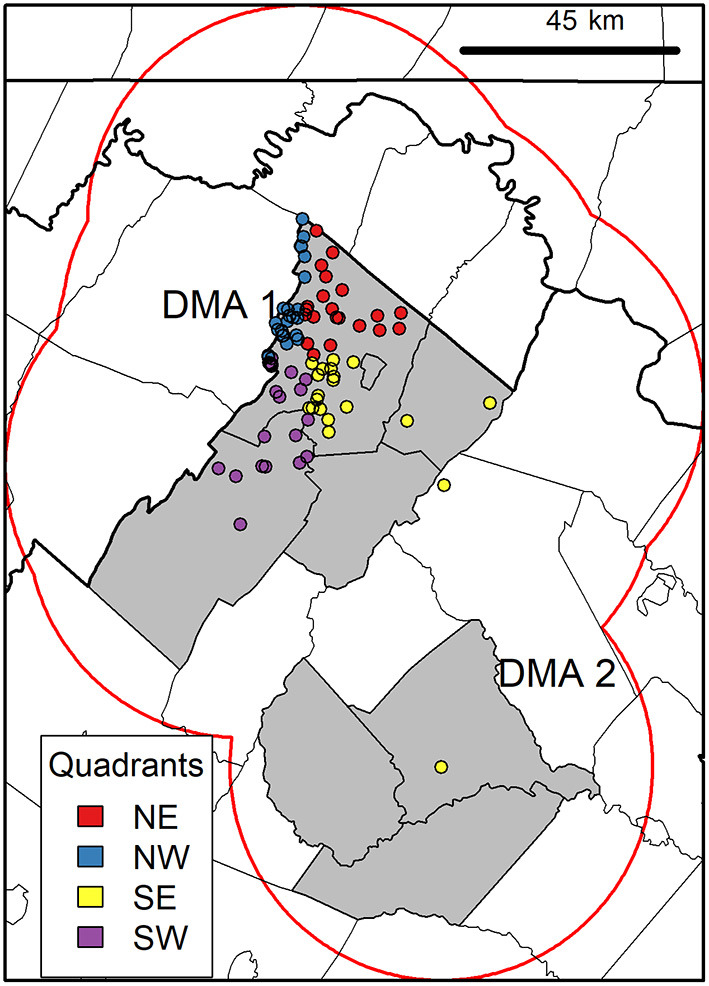
Case study area delineation and current CWD distribution. Map shows the case study area outline (gray rectangle) that was determined using dissolved buffers (red line) of maximum dispersal distance (45 km) (50) around positive cases (circles). Colored circles show quadrant organization of CWD positives (*n* = 88) used in modeling in Virginia Department of Wildlife Resources Disease Management Areas (DMA) 1 and 2 (gray polygons). This case study area was used for acquiring landscape information (see modeling workflow in Figure 3).

**Figure 3:**
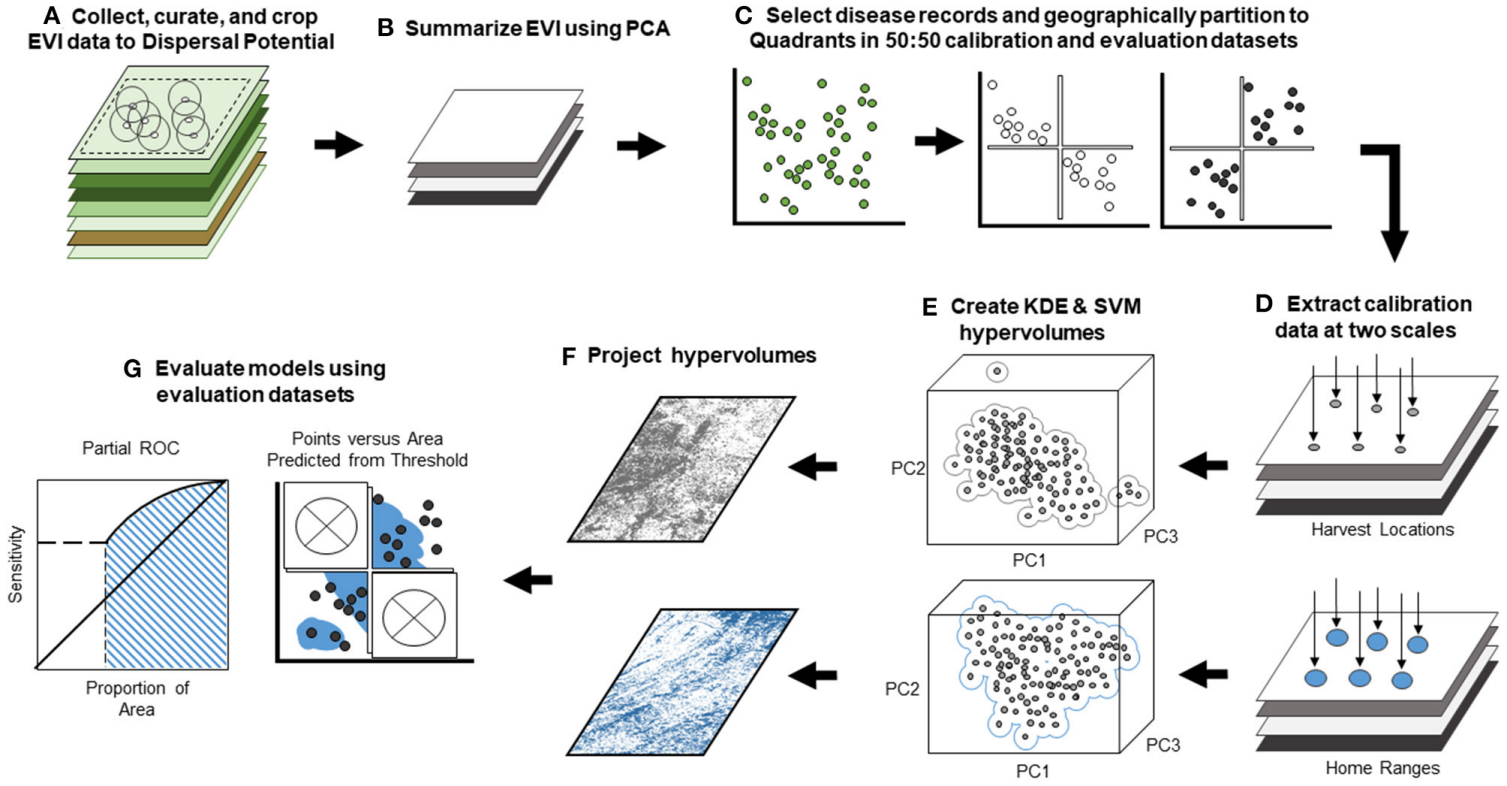
Workflow of black-box landscape epidemiology analysis. Workflow displays our black-box framework and evaluation procedure. **(A)** We collected remotely sensed enhanced vegetation index (EVI) and cropped rasters to the extent of the maximum dispersal potential for our focal species as radii about disease records (for details, see Figure 2). **(B)** We performed a principal component analysis on the EVI data to reduce multicolinearity and generate dimensions in analysis. **(C)** Next, we selected only disease occurrence locations and partitioned data into geographic quadrants for calibration and evaluation datasets. **(D)** We extracted principal component data from the PCA-generated raster stack at both *Harvest Location* (the single 250 m × 250 m raster pixel value corresponding to the precise sampling coordinate) and *Home Range* scales (a summary of values from multiple 250 m × 250 m pixels surrounding precise sampling coordinate) inferred from the home range size of focal species. **(E)** We developed 24 hypervolumes using Gaussian kernel density estimation and a one-class support vector machine for all six quadrant combinations and both scales. **(F)** Each hypervolume was projected onto geography in the form of binary risk maps, but KDE hypervolumes were additionally projected into continuous risk maps. **(G)** We used models to generate risk maps and evaluated models using methods appropriate for the projection: cumulative binomial probability testing (for binary maps) (69) and partial ROC (for continuous maps) (68). To more rigorously test models, we penalized suitability inherent to calibration data and restricted each map to evaluation dataset quadrants (represented by “×”).

Spatial models in general are affected by the study area extent, and should be meaningful in the context of the focal species (47). Thus, we confined the case study area based on the estimated movement potential (i.e., possible area accessible) of CWD reservoirs (48). More specifically, we used dispersal, or permanent movement away from a place of origin, due to its role in extreme bouts of deer movement (49). Because the maximum dispersal distance observed for white-tailed deer in Mid-Atlantic US is 45 km (50), we used this distance as a radius around CWD-positive cases, and took the dissolved union of circular buffers to define the extent of our rectangular study area (Figure 2). Next, we averaged individual 16-day EVI rasters to monthly pairs to reduce data gaps in grid cells caused by cloud obstruction and cropped rasters to the study area extent (Figure 3A).

We performed principal components analysis (PCA) on the EVI raster data (Figure 3B) to reduce multicollinearity and dimensionality in NicheA software (51). Principal component analysis ensures orthogonality in predictor variables by normalizing and transforming correlations found within data into new synthetic dimensions called principal components (PCs), which summarize both magnitude and direction of variance by generating eigenvalues and eigenvectors, respectively (52). Based on the long-term nature of our remote sensing data (i.e., 176 months of EVI data), our PCA generated 176 PCs, which were reduced to the first four statistically significant PCs—explaining 67% of total variance—*via* the broken-stick method (53). Significance was determined by whether the observed eigenvalues exceed those generated from null theoretical components (53–55). The first four PCs were used in our modeling as rasterized dimensions in environmental space.

### Epidemiological Data Preparation

Consistent with mentioned above, our black-box framework used a data-driven identification of landscape conditions occupied by confirmed CWD-positive cases (*n* = 88) in environmental space (56). To validate models, we divided data into calibration (model construction/training) and evaluation (model testing) sets in a 50:50 ratio (Figure 3C). We partitioned our data geographically rather than relying on random selection to avoid artificially inflating models' predictive performance (57). By partitioning CWD cases into geographic quadrants using their reported coordinates [i.e., 22 cases in each quadrant: northeast (NE), northwest (NW), southeast (SE), southwest (SW); Figure 2], we reduced spatial autocorrelation by ensuring spatial independence between quadrants used in calibration and evaluation (58). We tested all six combinations of paired quadrant arrangements in unique iterations of model calibration and evaluation.

We investigated two scales from which we extracted landscape data from the four stacked rasters from the PCA, referred to as *Harvest Location*^1^ and *Home Range* scales. For the *Harvest Location* scale, we extracted CWD-associated landscape data at the precise reported coordinates of CWD-positive deer (i.e., eigenvalues of the PCs found in the 250 × 250 m raster cell containing the coordinate) for *Harvest Location* models (Figure 3D). Yet, we assumed that simple harvest locations might fail to encompass the range of landscape conditions that motile white-tailed deer experienced, which could obscure CWD-landscape relationships. In the *Home Range* scale—named after the area most commonly inhabited for foraging, mating, and parental care (59)—we constructed buffers of 1.2 km^2^ which comprised multiple 250 m × 250 m raster cells surrounding the same coordinates of CWD cases to represent local home ranges (60) to capture a more generalized representation of landscape relationships (Figure 3D). Then, we averaged the PCA raster values found within the buffers constructed to generalize variation at a broader scale. We used these averages at each dimension as landscape data in *Home Range* models.

### Algorithm Selection and Model Calibration

We estimated the landscape conditions occupied by CWD-infected deer based on detailed delineation of environmental space occupied by cases (i.e., hypervolume), which can then be projected onto geography (61). Hypervolume estimation performance improves with use of a low number of continuous, uncorrelated variables to avoid constraining its shape (62), making our PCA landscape data preparation compatible with this algorithm selection in our black-box framework to identify areas of disease transmission risk using only positive cases. We determined the environments occupied by CWD cases using the *hypervolume* package in R (63, 64).

We used the PCA data extracted at our two scales (i.e., *Harvest Locations* and *Home Ranges*) from all six possible combinations of paired quadrants to be later evaluated with their complementary evaluation datasets (Figure 3D). To determine the environments occupied by cases, we developed hypervolume models using two algorithms for more robust analysis: Gaussian kernel density estimation (KDE) and one-class support vector machines (SVM) (65). Both algorithms for hypervolumes delineate environmental conditions where CWD transmission would be more likely, influenced by the parameters for each algorithm. In general, KDE uses kernel bandwidths, weighting of the data, and quantile thresholds to perform a density analysis in environmental space to delineate areas in the hypervolume model with higher probability given the data available (65). In contrast, SVM performs a cluster analysis to fit a boundary around data in environmental space that classifies conditions that should be similarly classified (i.e., “in” and “out” of the hypervolume), but potentially unobserved, requiring SVM to use smoothing parameter (γ) and error rate (ν) (65) (Figure 3E). Bandwidth selection in KDE determines how tightly the estimated probability density function fits the data in multivariate space (e.g., small bandwidth values yield high fit to the data). We followed previous efforts supporting the use of smoothed cross validation to determine KDE bandwidth for four dimensional data (66), which also has been reported to reduce predictive error in hypervolumes (65). Additionally, based on the comprehensive surveillance of DWR, we allowed even weighting of the data because we assumed each CWD case was equally probable in describing environmental conditions. We assumed a consistent quantile threshold of 95% (α = 0.05) to curtail the KDE probability density to give the hypervolume its shape (2, 65). Furthermore, we relied on the default SVM parameters of γ = 0.5 and ν = 0.1, based on their support found in literature (65). Finally, we projected each of the 24 hypervolumes generated from all quadrant combinations, algorithms, and scales (i.e., six quadrant combinations at two scales for two algorithms) from environmental space onto geographic space (Figure 3F) in the form of risk maps for CWD transmission to evaluate models.

### Model Evaluation

As mentioned above, model evaluations are needed to determine the predictive abilities of algorithms in a black-box framework. We evaluated predictive abilities in the 24 hypervolumes by testing the hypothesis that, when projected in the form of risk maps, hypervolume models are predicting CWD transmission in landscapes that were independent of model calibration (i.e., evaluation quadrants) better than a random expectation. For example, when the NE and SW quadrants were used for model calibration, the NW and SE quadrants were used for evaluation, and model predictions would be deemed statistically significant if risk maps for evaluation quadrants appropriately predict risk where known CWD cases have occurred (adopting α = 0.05) (Figure 3G). We restricted risk map projections to the quadrants independent of model calibration because model evaluation methods that rely on the quantification of the proportion of areas predicted as “suitable” for high risk (67) would be inherently inflated in model calibration quadrants.

Evaluation methods were specific to type of geographic map generated when projecting hypervolumes. Binary outputs (i.e., no risk = 0, risk = 1), for example, were the only option in SVM-delineated hypervolumes due to the nature of classification; however, we selected a fixed 95% threshold to generate binary maps for hypervolumes delineated with KDE. For all binary maps, we used a cumulative binomial probability distribution accounting for the proportion of area predicted as “suitable” for risk and the number of independent occurrence records successfully being predicted by the map (2, 68). For model projections of continuous probability from KDE, we used the partial receiver operating characteristic (partial ROC) in the *ntbox* RShiny application (69). Unlike more common ROC and AUC evaluations dependent on presence/absence data, partial ROC evaluates the relationship between model sensitivity in relation to varying thresholds of proportional area predicted with a user-defined error rate assumed from false negatives (67). Specifically, we used 500 bootstrapping samples using 50% of the complementary evaluation data of the models resampled with replacement, accounting for a 5% error rate in omission presumed from any errors in diagnostic methods. In partial ROC, model evaluation interpretations are based on the ratio between the model's area under the partial ROC curve and a null model, whereby ratio values >1 suggest model performance in predicting independent data is better than a random expectation (67). Furthermore, each bootstrap iteration resampling of the evaluation data yields an AUC ratio; model performance is determined to be better than random, and the null hypothesis is rejected when ≥95% of the samples in this distribution are >1 [for further clarification, software and example data, please see (70)]. We compared predictive abilities between scales by performing paired *t*-tests on the resampled AUC ratio distributions, which allowed us to capture uncertainty among a population of significance levels.

### Uncertainty Estimation

To examine whether CWD occurred in consistent and quantifiable vegetation phenology conditions, we examined variation of hypervolume models from different data availability scenarios as a proxy of uncertainty (55, 71, 72). More specifically, we generated models with different magnitudes of CWD data to determine whether CWD occurred in consistent environmental conditions. This consistency was determined by measuring the change in position and size of hypervolume models relative to changes in CWD data. For both scales (*Harvest Locations* and *Home Ranges*), we used an approach resembling a jackknife (i.e., leave-one-out) by building multiple hypervolume models iteratively removing single occurrence records (i.e., *n* – 1), which is generally used in statistics to assess model variation (52, 58) (Supplementary Figure 1A). To assess variation in hypervolume models, we compared all leave-one-out hypervolume models against a model using the full CWD dataset as a baseline. In other words, a weak similarity (overlap) in the position and size between a leave-one-out hypervolume to the baseline would indicate the removed record had strong influence in describing phenology conditions for the baseline model. These similarity metrics were determined using the Jaccard similarity index, which calculates intersection of two hypervolume models (i.e., full data vs. leave-one-out) relative to their union, where values of 0 indicate dissimilar models and 1 indicate identical models (73) (Supplementary Figure 1B). Also, because similarity metrics depend on their volume occupied in environmental space, we examined variation in hypervolume model size by calculating the volume for each leave-one-out hypervolume. By assembling leave-one-out hypervolume models *via* averaging, we generated single continuous risk maps at each scale that identify areas where high-risk predictions were consistent among all data availability scenarios for DWR use. Finally, we explored differences in EVI signatures in consistent high risk areas (i.e., areas where more than half of the leave-one-out models identified probable risk) and less consistent risky areas (i.e., less than half of the leave-one-out models identified probable risk) by comparing mean and standard deviation of EVI over time.

## Results

### Model Evaluation Results

**Figure 4:**
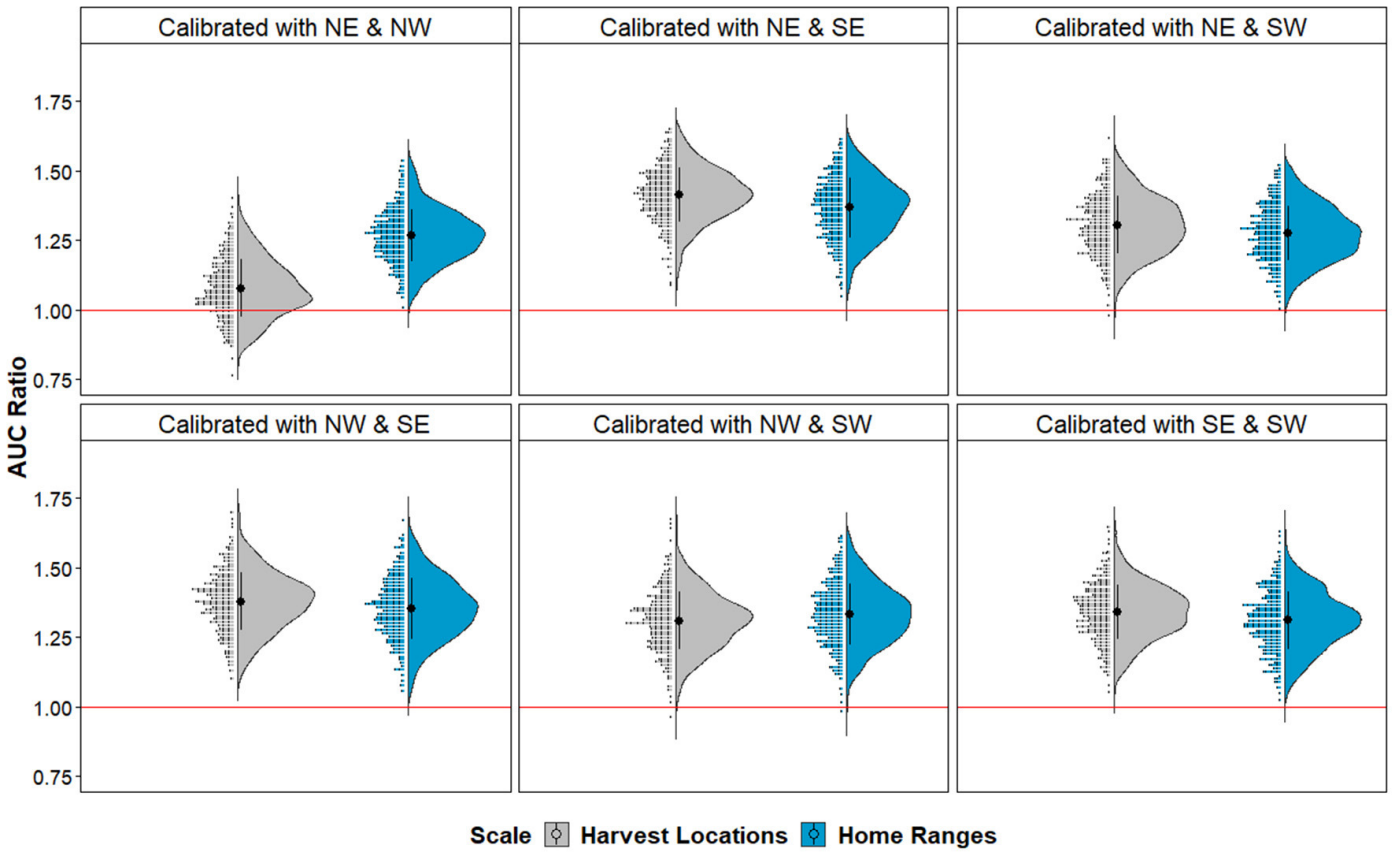
AUC ratio evaluation from partial ROC of continuous suitability maps in each geographic partition. Model evaluation according to different quadrants configuration used for calibration. Half-violin and raw data distribution plots denote bootstrapped AUC ratios obtained from the evaluation quadrants (not used in model calibration) for models based on *Harvest Locations* (gray) and *Home Ranges* (blue). Note that most configurations have AUC ratios >1, which is above the threshold for random expectation (red line; *p* < 0.001), except for one *Harvest Location* model calibrated with the northeast and northwest quadrants with non-significant predictions (*p* > 0.05). Ribbon abbreviations follow cardinal directions (i.e., NE, northeast; NW, northwest; SE, southeast; SW, southwest).

Upon evaluation of the algorithms used in our black-box framework, we found that both KDE and SVM algorithms generated similarly statistically significant predictions of CWD cases according to cumulative binomial probability testing (Table 1). That is, for all iterations of different quadrant combinations, model-generated binary maps predicting CWD risk identified the areas where CWD-positive cases which were independent from model calibration better than random. Notably, models were statistically significant at both scales despite the proportion of area projected as suitable being higher in hypervolumes delineated from *Harvest Locations* (Table 1). Similarly, when evaluating KDE-delineated hypervolume projections of continuous risk outputs, partial ROC and bootstrapping manipulations identified that most data resampling resulted in models with AUC ratios >1 in all paired quadrant combinations signifying predictive abilities clearly better than random with respect to the estimated uncertainty (Figure 4). Hypervolume models calibrated from *Home Ranges* yielded significantly higher AUC ratios to those from *Harvest Locations* [μ = 1.318 and 1.305, respectively; *t*_(5, 531)_ = 3.949, *p* < 0.001]. The lowest AUC ratios observed occurred in the iteration where the landscape conditions from CWD-positives in the NE and NW quadrants at the *Harvest Locations* scale were used in calibration. This iteration still had a mean AUC ratio >1 (μ = 1.1), but as a whole did not predict better than random expectation (*p* > 0.05) as >5% of the AUC ratios from bootstrapping were <1 (Figure 4).

**Table 1:** Evaluation of binary suitability maps generated from both algorithms under all quadrant and scale combinations.

**Calibration quad**.	**Evaluation quad**.	**Scale**	**Successes**		**Trials**	**Proportion of suitable area**		***p*-value**	
			**KDE**	**SVM**		**KDE**	**SVM**	**KDE**	**SVM**
NE and NW	SE and SW	Harvest	38	19	44	0.675	0.167	0.001	<0.001
NE and SW	NW and SE	Harvest	40	21	44	0.707	0.251	<0.001	<0.001
NE and SE	NW and SW	Harvest	42	17	44	0.711	0.234	<0.001	0.007
NW and SW	NE and SE	Harvest	38	27	44	0.767	0.260	0.038	<0.001
NW and SE	SW and NE	Harvest	41	21	44	0.715	0.208	<0.001	<0.001
SW and SE	NW and NE	Harvest	44	22	44	0.710	0.289	<0.001	<0.001
NE and NW	SE and SW	Range	35	10	44	0.458	0.070	<0.001	<0.001
NE and SW	NW and SE	Range	33	15	44	0.527	0.147	0.001	<0.001
NE and SE	NW and SW	Range	34	13	44	0.495	0.127	<0.001	<0.001
NW and SW	NE and SE	Range	36	20	44	0.599	0.176	0.001	<0.001
NW and SE	SW and NE	Range	34	12	44	0.443	0.111	<0.001	<0.001
SW and SE	NW and NE	Range	34	13	44	0.467	0.133	<0.001	0.001

*Cumulative binomial testing [following (68)] use the number of successes (i.e., number of coordinates of CWD cases that successfully occur within modeled suitable landscapes for transmission risk), trials (i.e., total number of coordinates of CWD cases being tested from quadrants found in “Testing Quad”), and proportion of the area suitable for transmission risk relative to the overall area to generate significance levels (i.e., p-values). Results from models that were delineated using two algorithms: kernel density estimation (KDE) and one-class support vector machines (SVM) can be found within their respective columns. Note that all combinations of quadrants (rows) from data partitioning yielded statistically significant predictions better than by random expectation (p <0.05). Scale specifies whether models were delineated from data at Harvest Locations (HARVEST) or Home Ranges (RANGE) (for explanation, see Figure 3)*.

### Uncertainty Estimation

**Figure 5:**
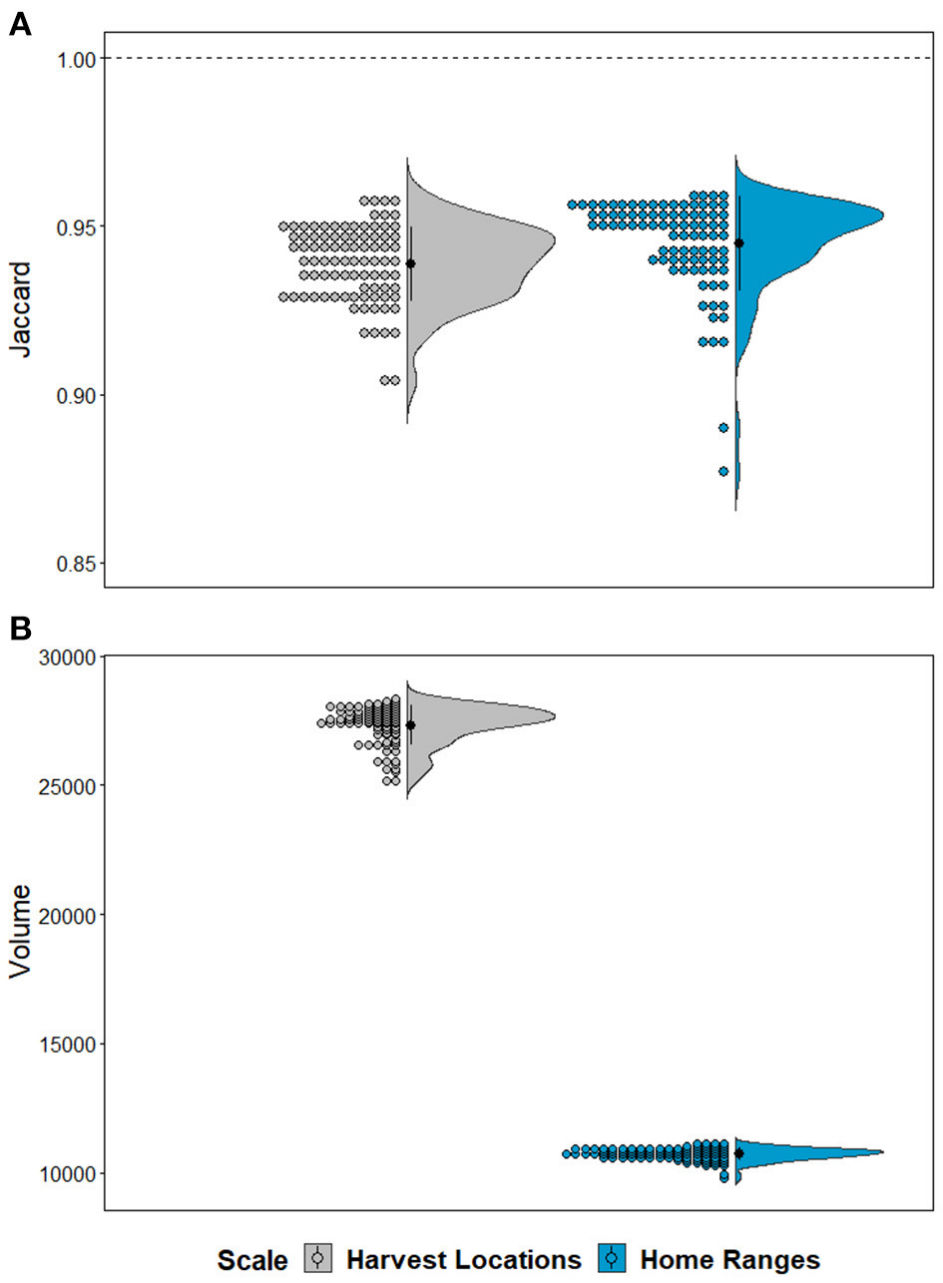
Hypervolume variation and characteristics by scale. **(A)** Plots show Jaccard's similarity index between hypervolume sets of the full CWD-positive dataset (*n* = 88) and those created from iteratively removing one occurrence record (leave-one-out). Note that the models never reach a Jaccard similarity index values at 1 denoted with dashed horizontal line, which indicates a failure to reach complete overlap and identical position and size in environmental space. **(B**) Half-violin plots and raw data distribution represent volumes extracted from hypervolumes created from leave-one-out iterations. Colors represent the scale for whether models were delineated from data at *Harvest Locations* (gray) or *Home Ranges* (blue). Note that hypervolumes from home ranges generally occupied smaller volumes in environmental space despite equal sample sizes.

**Figure 6:**
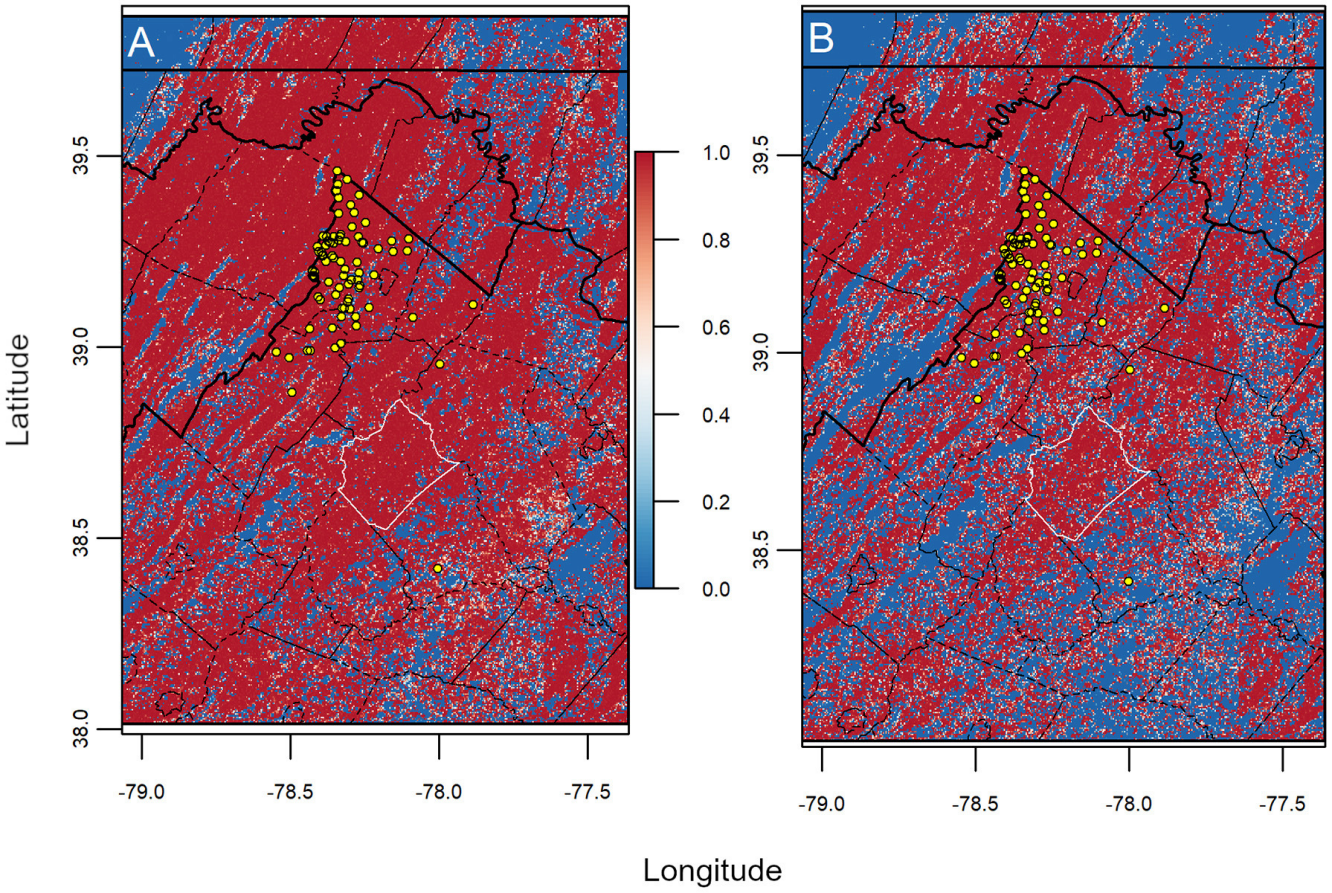
Maps of projected CWD transmission risk from uncertainty analysis. Risk maps identify areas determined with more consistent probable risk (red) and less consistent risk (blue) for CWD transmission from jackknife analysis. We found more homogenous and widespread transmission risk being consistent among models using **(A)** *Harvest Locations* compared with **(B)** *Home Ranges*. Note that counties with considerable transmission risk include Rappahannock County (white outlined polygon). Lines indicate boundaries of states (thick black) and counties (thin black), while points (yellow circles) represent known CWD cases (*n* = 88). Overall, the amount of area predicted as consistently risky was higher in models generated from *Harvest Locations*.

We found considerable overlap in environmental space, represented in Jaccard similarity among hypervolume models with different magnitudes of CWD data (μ = 0.94 for *Harvest Locations* and μ = 0.95 for *Home Ranges;* Figure 5A). Still, Jaccard values of overlap were variable among iterations of data availability scenarios (i.e., *n* – 1) both within and between scales, where Jaccard values from *Home Ranges* were significantly higher than *Harvest Locations* [*t*_(87)_ = 3.632, *p* < 0.001]. We also observed differences in calculated volumes in environmental space (Figure 5B). Size (i.e., volume) from models built using *Home Ranges* were generally smaller in volume [*t*_(87)_ = −246.38, *p* < 0.001] compared with *Harvest Locations* (Figure 5B). Next, maps assembled from the leave-one-out hypervolume models revealed areas of consistently predicted CWD transmission risk was heterogeneous across the study area and between scales (Figure 6). In general, mapped hypervolume models delineated with data at *Harvest Locations* predicted CWD transmission risk across broader extents of the study area (Figure 6). From our exploratory analysis of EVI, we identified nearly identical mean EVI values between areas predicted with consistent high risk and those with less consistent risk, but we found generally higher mean EVI values during the 129 Julian day (early May) and lower standard deviation in EVI in areas with consistent CWD transmission risk throughout most of the year (Supplementary Figure 2).

## Discussion

Predicting where wildlife diseases may occur next is a challenging pursuit that relies on careful collection of predictor variables, epidemiological data, and consideration of the ecology of the host species. Here, our black-box framework demonstrated that using remotely sensed vegetation phenology data alone can predict CWD transmission risk with statistical significance, suggesting EVI could serve as another tool for predicting CWD distributions in early outbreaks. Furthermore, we highlighted that consideration of the ecology of the host species represented through a home range can enhance understanding for a free-ranging wildlife disease.

By using a method that accounts for independent evaluation data and the area predicted with respect to the area available (i.e., AUC ratio), we found quantitative support for the use of landscape information to trace CWD transmission risk. We quantified the extent to which CWD could be reliably predicted on the landscape using our data-driven hypervolume models delineated with both KDE and SVM. This predictive ability was determined using the proportion of areas predicted as risky under both binary and continuous risk projections. We found that *Home Range* models that acknowledged the host species' ecology generated significantly different outcomes in performance via AUC ratios than those developed from landscape determinants at *Harvest Locations*. Specifically, *Home Range* models yielded higher AUC ratios. We suspect this finding is either a result of summarizing the heterogeneity in EVI surrounding each CWD case in a framework compatible with the landscape ecology of chronic diseases or compensates for any discrepancies in spatial uncertainty related to hunting grid resolution.

In the context of CWD in Virginia, our jackknife analysis identified that every CWD case influences the amount of risk predicted. Hypervolumes never reaching a complete overlap (i.e., a Jaccard index value of 1) could suggest that variation seen in environmental space with new CWD cases could stem from disease non-equilibrium, meaning CWD current distribution may not be exhausting its potential occupancy of environmental conditions (74). Under this finer-population scale, this would not be surprising given what is known about nearby CWD cases outside of Virginia withheld from this analysis, the range of landscape conditions that CWD has been identified worldwide (e.g., Scandinavia) (75), and the environmental hardiness of prions in general. Results from our uncertainty analysis identify that landscape conditions associated with consistent high CWD transmission risk have been observed consistently in portions of DMA1 and DMA2, where DWR has conducted comprehensive sampling.

Consistently high-risk areas distant from known CWD cases could suggest new landscapes for potential CWD establishment assuming host dispersal is plausible, though human-associated movement of infected cervids or tissues still threaten unpredicted areas (13). Notably, we identified EVI variation associated with consistent high CWD risk in Rappahannock County, which remained outside disease management area delineation during our study period (harvest seasons 2009–2019), and thus has seen historically lower surveillance effort relative to neighboring counties within DMAs (μ = 6 samples/year from 2007 to 2019). In light of these results, we would suggest that increased surveillance during future harvest seasons with selective removal of high-risk demographic groups or promoting convenience sampling (e.g., roadkill deer) in consistently risky counties could be prudent for management consideration and narrowing surveillance within the geographic extent of the predicted risk area (34, 76); the former action would also remain predicated on public support considering its potential controversy among hunters. We found some differences in EVI between consistent high-risk and less consistent risk areas. Relative to less consistent risk areas, consistent high-risk areas had marginally lower variation in EVI in the late winter and higher mean EVI values in the period in later spring. These periods are consistent with the time of year corresponding to green-up and peak vegetative maturity following green-up, respectively (77). Although formal descriptive analysis to understand the mechanisms of green-up and how it relates to CWD extended beyond the scope of our predictive analysis, the avenue of research appears important given what is known about how green-up relates to ungulate behavior and biology (78, 79) and inspires future research directions.

The strength of black-box approaches lies in their nature of modeling the disease outbreaks *sensu stricto*, which can elucidate landscape relationships for poorly known diseases in humans and animals. Critical starting points in landscape epidemiology of orthopoxviruses and filoviruses (31, 80) have relied on black-box frameworks to support public health interventions, for example. Still, despite the unclear ecology of prions in the environment relative to other pathogens (9), we found analysis of CWD outbreaks were surprisingly withheld from black-box frameworks. This gap is likely related to our team's finding that landscape ecological approaches in general have been less common relative to population-level models in CWD research (6). In Virginia, our use of EVI identified and predicted CWD distribution using relatively few samples. Our approach might be particularly constructive for elucidating high-risk areas in newly established outbreaks when combined with weighted surveillance strategies (20, 81), although identifying the minimum sample size required with our approach remains beyond the scope of our analysis. Through our work here, we provide evidence for a complementary method that may precede more complex, descriptive models focused on comparing relative influences of specific landscape risk factors (e.g., logistic regression models, Bayesian hierarchical models, and generalized linear models) (21, 82, 83) that may depend on large sample sizes or well-informed prior inferences (84). We further support the value of *n*-dimensional hypervolumes in this landscape ecological and landscape epidemiological research, which is a subject area surprisingly withheld from the technique to our knowledge, despite hypervolume's functionality in ecological modeling of species distributions and niches (61).

Our work presents methodology that is novel to CWD and prion diseases in general. Yet, we recognize some inherent limitations to our modeling. For example, hypervolume models are data driven, therefore additional data from new CWD cases could identify different patterns. Similarly, because of the nature of black-box approaches, predictors could estimate risk differently in different areas, but more research is needed to understand mechanisms related to these differences. Relative to other algorithms (i.e., Maxent), hypervolume algorithms also do not provide strong extrapolation capabilities to areas outside of the study area. Nevertheless, our methodology selection permits a reduced number of parameters and their respective assumptions. Furthermore, our data are reliant on diagnostic tests with sensitivity noted for false negatives (39); however, the current data yield patterns that can facilitate management decisions and emphasize the strength of our presence-only modeling protocol. We recognize the assumption of inferring home range sizes surrounding CWD cases for model construction may be simplistic relative to empirical data (e.g., GPS-collaring cervids, integrating demographic differences in home ranges). Clearly, such data demand resources, logistics, and ethical considerations that may be prohibitive, sensitive to seasonality, restricted to finer-scale landscapes, and contradictory to management objectives (e.g., permitting CWD-infected cervids on the landscape to understand changes in home range sizes and dispersal) (85).

Past CWD landscape ecology research utilized numerous, often static data sources for associating disease risk factors (e.g., national land cover datasets, human population densities) (6), which generate useful results for descriptive modeling. Our predictive case study serves to utilize a dataset spanning over one decade with corresponding moderate spatial and temporal resolution remote sensing data to predict CWD transmission risk among dimensions of variation in environmental predictors. Our finding that model performance improved from testing broader-scale landscape conditions associated with a chronic and cryptic disease represented as conditions present in potential home ranges suggests an avenue for future research. For instance, the occurrence of spatial “outlier” cases in CWD epidemics could possess similar spatiotemporal conditions in environmental space, or landscape models calibrated from harvest locations may harbor bias in landscape relationships similarly found in population-level studies (e.g., prevalence estimates) (35). We show here that CWD was able to be mapped using tools effective for other organisms, even though CWD is caused by a very poorly known pathogen (86). Indeed, until unified protocols to study the landscape epidemiology of prion diseases are identified or even possible to be generalizable, previous approaches have demanded large, often prohibitive sample sizes to identify relationships between CWD-positive and CWD not-detected cervids. If modeling intentions are strictly predictive, our project provides evidence of the capacities of widely available and standardized satellite-derived landscape data to reconstruct CWD transmission risk in free-ranging populations under natural conditions.

## Data Availability Statement

The datasets presented in this article are not readily available. Restrictions apply to the data used in the article and so it is not publicly available, but was used under license for the current study. The data will be made available from the authors upon request and with permission from Megan S. Kirchgessner, DVM, PhD (megan.kirchgessner@dwr.virginia.gov, DWR). We've supplied a sample R code of the analyses and the artificial data within our study area for transparency on a public GitHub repository (https://github.com/SNWinter/CWD_Landscape_VA).

## Author Contributions

SW contributed to the design of the project, performed the data collection, data analyses, and wrote the manuscript. MK provided epidemiological data and subject area expertise, performed editions of manuscripts, and wrote the manuscript. EF provided statistical expertise, performed editions, and wrote the manuscript. LE contributed to the design of the project, supervised the methodology, and wrote the manuscript. All authors contributed to the article and approved the submitted version.

## Conflict of Interest

The authors declare that the research was conducted in the absence of any commercial or financial relationships that could be construed as a potential conflict of interest.

## Publisher's Note

All claims expressed in this article are solely those of the authors and do not necessarily represent those of their affiliated organizations, or those of the publisher, the editors and the reviewers. Any product that may be evaluated in this article, or claim that may be made by its manufacturer, is not guaranteed or endorsed by the publisher.

## Footnotes

^1^Please note that the “*Harvest Location*” term simply describes the most precise known location of sampled deer. The term was adopted because most cases originated from hunter harvest, but in our use also includes positive cases identified using other sampling methods (e.g., clinical sign euthanasia).
