# Supplementary material for: A Landscape Epidemiological Approach for Predicting Chronic Wasting Disease: A Case Study in Virginia, US
Source: Front Vet Sci. 2021 Aug 24;8:698767. doi: 10.3389/fvets.2021.698767 (PMC8421794; doi:10.3389/fvets.2021.698767)
Supplement: Supplementary file 1 [file Data_Sheet_1.pdf]

Supplementary Material

A landscape epidemiological approach for predicting chronic wasting disease: A case study in Virginia, US

Steven N. Winter[1], Megan S. Kirchgessner[2], Emmanuel A. Frimpong [1], and Luis E. Escobar[1*]

[1]Laboratory of Disease Ecology and Biogeography, Department of Fish and Wildlife Conservation, Virginia Polytechnic Institute and State University, Blacksburg, VA, USA

[2]Virginia Department of Wildlife Resources, Blacksburg, VA, USA

*** Correspondence:**
Corresponding Author
escobar1@vt.edu

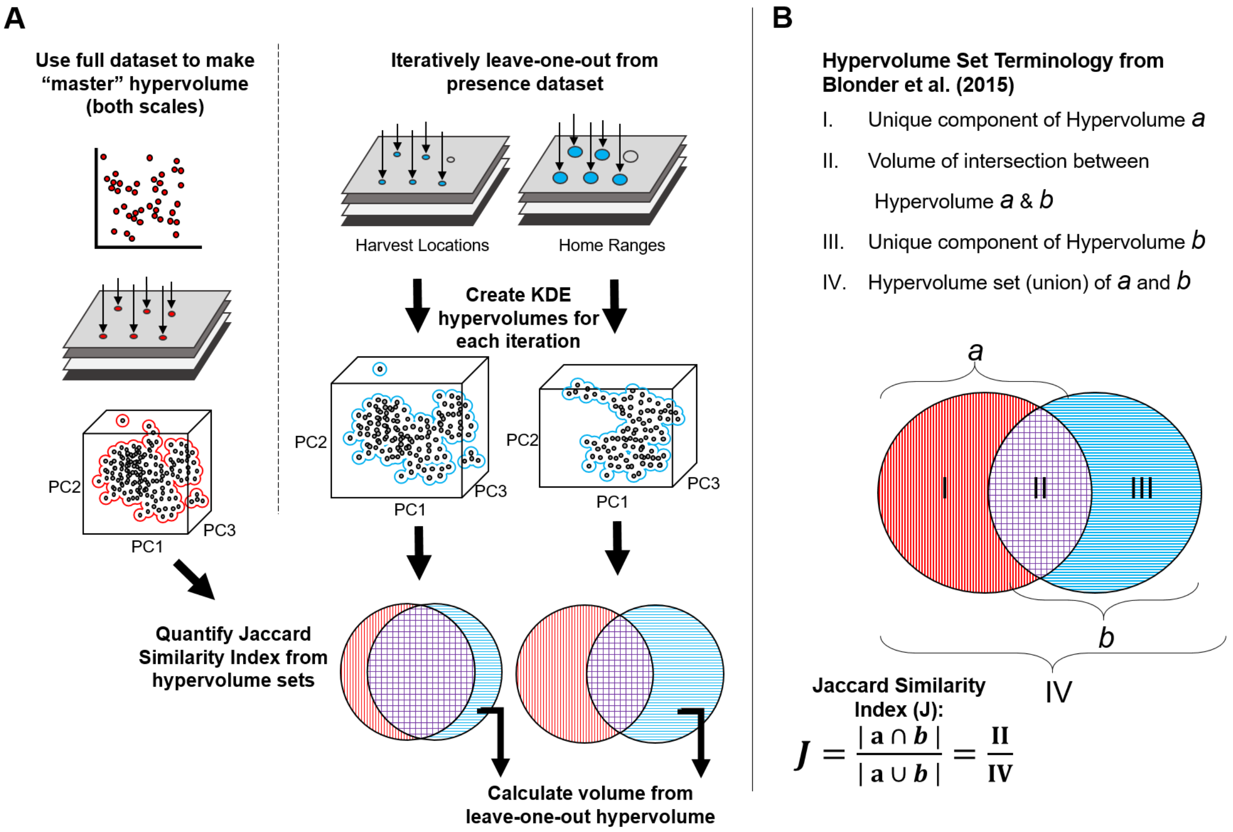

Supplementary Figure S1: Workflow for hypervolume uncertainty analysis. **A**) Environmental variation determined by iteratively removing one CWD-positive case from total dataset, creating KDE hypervolumes for each iteration and examining overlap between total dataset (n=88) and subset

(*n*=87). **B**) Key to terminology and equations used in calculating hypervolume overlap statistics including components that comprise Jaccard similarity index, adapted from (1).

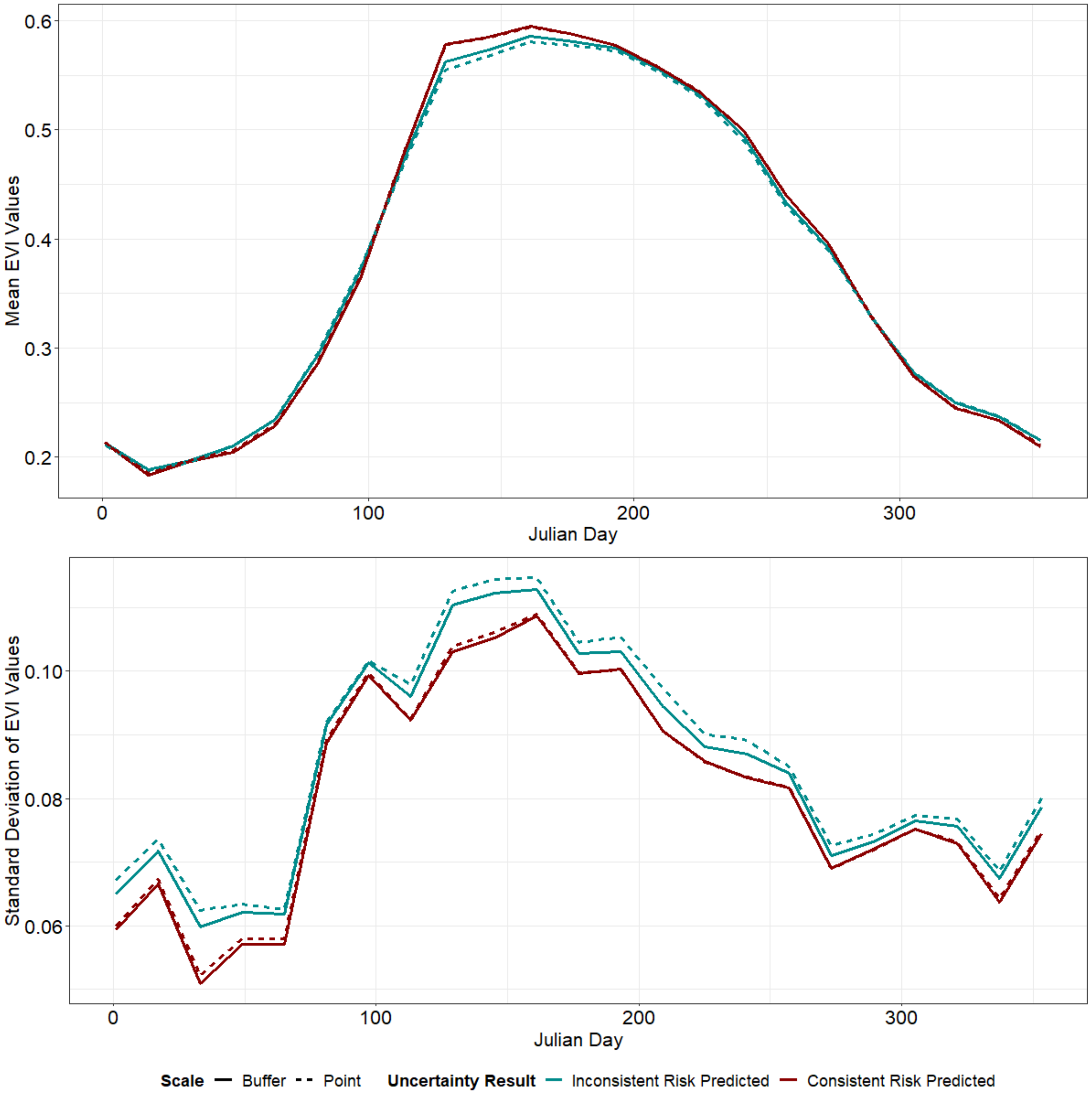

Supplementary Figure S2: Descriptive results of annual EVI conditions from 2005-2019 from projected risk maps in uncertainty analysis. We used the projected risk maps from Fig. 6 to explore differences of EVI at grid cells predicted with high consistent risk (red lines), and those predicted with less consistent risk (blue lines) for projected risk maps made from hypervolume models at each scale (i.e., Home Range buffers as solid lines and Harvest Location points as dashed lines). Mean EVI values (top panel) were largely consistent across scales and risk prediction groups, but consistent, high risk areas observed higher mean EVI values noted in early May (approximately near Julian day 129). Standard deviation of EVI values were lower in high consistent risk areas in late winter (approximately near Julian day 38-65).

1 References

1. Blonder B. hypervolume: High dimensional geometry and set operations using kernel density estimation, support vector machines, and convex hulls. (2019) R package.
